# DISC1 overexpression promotes non-small cell lung cancer cell proliferation

**DOI:** 10.18632/oncotarget.18055

**Published:** 2017-05-22

**Authors:** Shuo Wang, Ying-Ying Chen, Yu-Peng Li, Jun Gu, Shu-Dong Gu, Hai Shi, Xue-Song Li, Xiao-Ning Lu, Xiang Li, Shuang-Long Zhang, Kang-Jun Yu, Kun Liu, Li-Li Ji

**Affiliations:** ^1^ Department of Cardiothoracic Surgery, Affiliated Hospital of Nantong University, Nantong, China; ^2^ Department of Pathology, Medical School of Nantong University, Nantong, China; ^3^ Department of Immunology, Medical School of Nantong University, Nantong, China; ^4^ Department of Oncology, Affiliated Hospital of Nantong University, Nantong, China; ^5^ Department of Pediatrics, The People’s Hospital of Rizhao, Rizhao, China; ^6^ Department of Respiratory, Affiliated Hospital of Nantong University, Nantong, China; ^7^ Department of Cardiothoracic Surgery, The Third People's Hospital of Nantong, Nantong, China; ^8^ Department of Otorhinolaryngology, Maternal and Child Health Care Hospital of Nantong, Nantong, China

**Keywords:** non-small cell lung cancer, DISC1, GSK3β, β-catenin, proliferation

## Abstract

Neuropsychiatric disorder-associated disrupted-in-schizophrenia-1 (DISC1) activates Wnt/β-catenin signaling by inhibiting glycogen synthase kinase 3 beta (GSK3β) phosphorylation, and may promote neural progenitor cell and pancreatic β-cell proliferation. The present study found that DISC1 promotes non-small cell lung cancer (NSCLC) cell growth. Western blotting and immunohistochemistry analyses showed that DISC1 was highly expressed in NSCLC cell lines and patient tissues. DISC1 expression was negatively associated with phosphorylated (p-) GSK3β, but positively correlated with a more invasive tumor phenotype and predicted poor NSCLC patient prognosis. siRNA-mediated DISC1 silencing increased p-GSK3β expression and decreased expression of β-catenin and Cyclin D1, while DISC1 upregulation produced the opposite results. DISC1 knockdown also reduced NSCLC cell proliferation rates *in vitro*. These results suggest that DISC1 promotes NSCLC growth, likely through GSK3β/β-catenin signaling, and that DISC1 may function as an oncogene and novel anti-NSCLC therapeutic target.

## INTRODUCTION

Lung cancer is the most frequently occurring cancer type, and the leading cause of cancer death globally [1, 2]. Most lung cancer cases are diagnosed at an advanced stage, and approximately 85% are non-small cell lung cancer (NSCLC) [1, 3]. NSCLC patient prognosis remains poor; five-year disease-specific mortality rates for stages I–IIIA are 30%, 60%, and 75%, respectively [4]. New diagnostic markers are urgently needed to enhance early lung cancer detection and targeted treatment.

Disrupted-in-schizophrenia-1 (DISC1) is a risk gene associated with major mental illnesses, including schizophrenia and depression [5]. A balanced t[1; 11] [q42.1; q14.3] translocation that disrupts DISC1 co-segregates with schizophrenia, major depression, and bipolar disorder [6–8]. DISC1 may regulate progenitor cell proliferation and neuronal migration processes [9, 10]. DISC1 reportedly activates Wnt/β-catenin signaling by inhibiting glycogen synthase kinase 3 beta (GSK3β) kinase activity, thus increasing β-catenin protein abundance and transcriptional activity [11–13].

The canonical Wnt/β-catenin pathway modulates cell proliferation, which is negatively regulated by the β-catenin destruction complex. GSK3β promotes β-catenin phosphorylation, resulting in β-catenin ubiquitylation-mediated degradation in the proteasome [14–18]. However, when β-catenin accumulates in the cytoplasm, it translocates to the nucleus and activates target genes involved in cell proliferation, including Cyclin D1 and c-Myc [19]. Aberrations in this pathway are implicated in the pathogenesis of multiple tumor types, including lung cancer [20–22].

The present study assessed DISC1 expression in human NSCLC cell lines and tumor tissues, and evaluated relationships between DISC1 expression, clinicopathological features, and patient prognosis. We also investigated the role of DISC1 in cancer cell proliferation. We demonstrated that high DISC1 expression promotes NSCLC growth, possibly through GSK3β/β-catenin signaling.

## RESULTS

### DISC1 expression in human NSCLC cell lines and tissues

**Figure 1:**
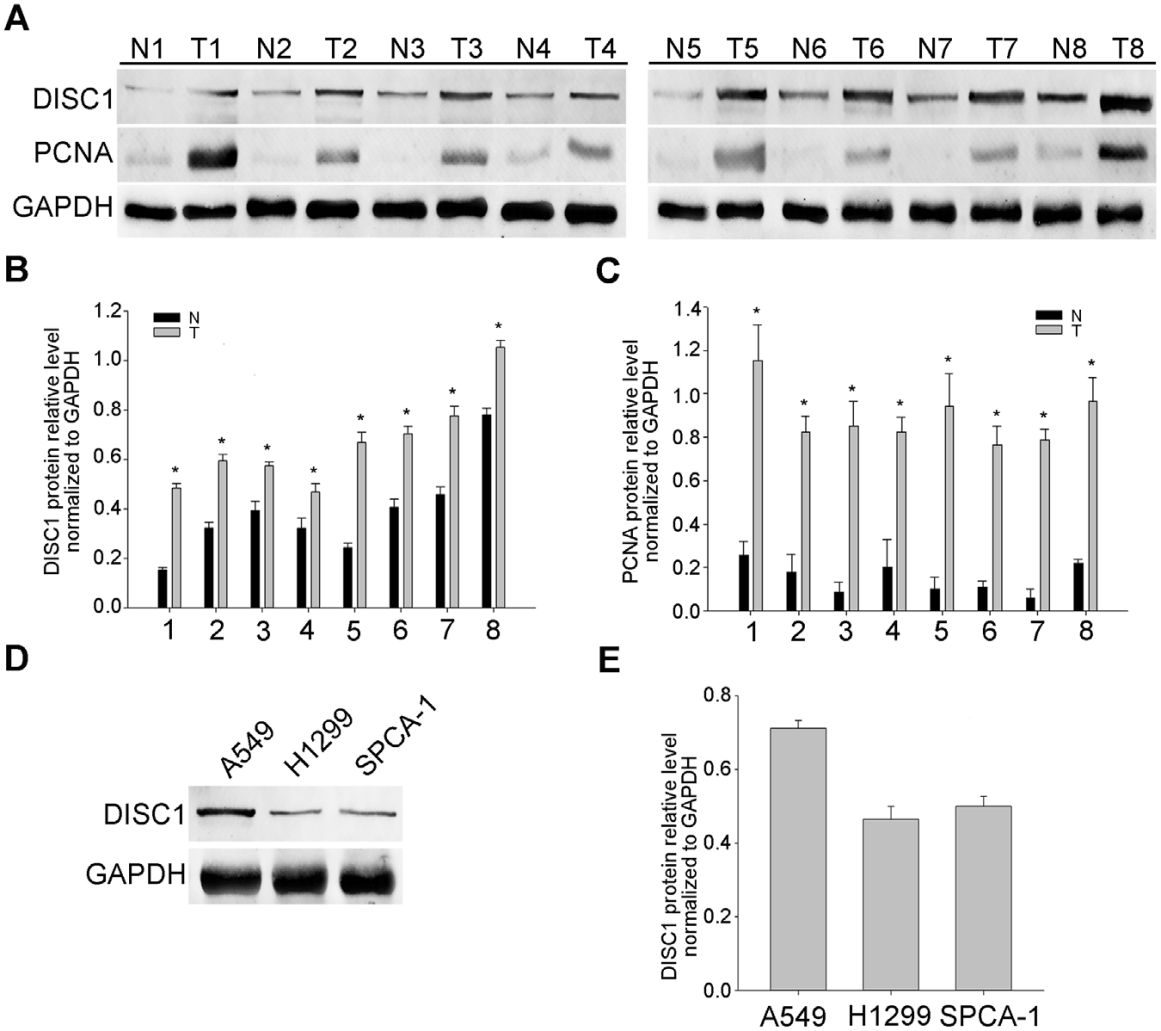
DISC1 expression in human NSCLC tissues and cell lines DISC1 and PCNA levels in eight paired NSCLC tumor (T) and adjacent non-tumor (N) tissues as shown by western blot analysis **(A)** GAPDH was used as a loading control. DISC1 **(B)** and PCNA **(C)** levels were analyzed in tumor tissues (normalized to GAPDH). *P<0.05, compared with adjacent non-tumor tissues. DISC1 expression in NSCLC cell lines (A549, H1299, and SPCA-1) as shown by western blot analysis **(D)** GAPDH was used as a loading control. DISC1levels were analyzed in NSCLC cell lines (normalized to GAPDH) **(E).**

Western blotting results showed that DISC1 and PCNA (a cancer cell proliferation marker) levels were higher in NSCLC tissues than in paired adjacent non-tumor tissues (Figure 1A–1C), and exhibited similar expression patterns. DISC1 was also highly expressed in the NSCLC cell lines, A549, H1299, and SPCA-1 (Figure 1D–1E).

### DISC1 expression is correlated with NSCLC patient clinicopathological parameters

**Figure 2:**
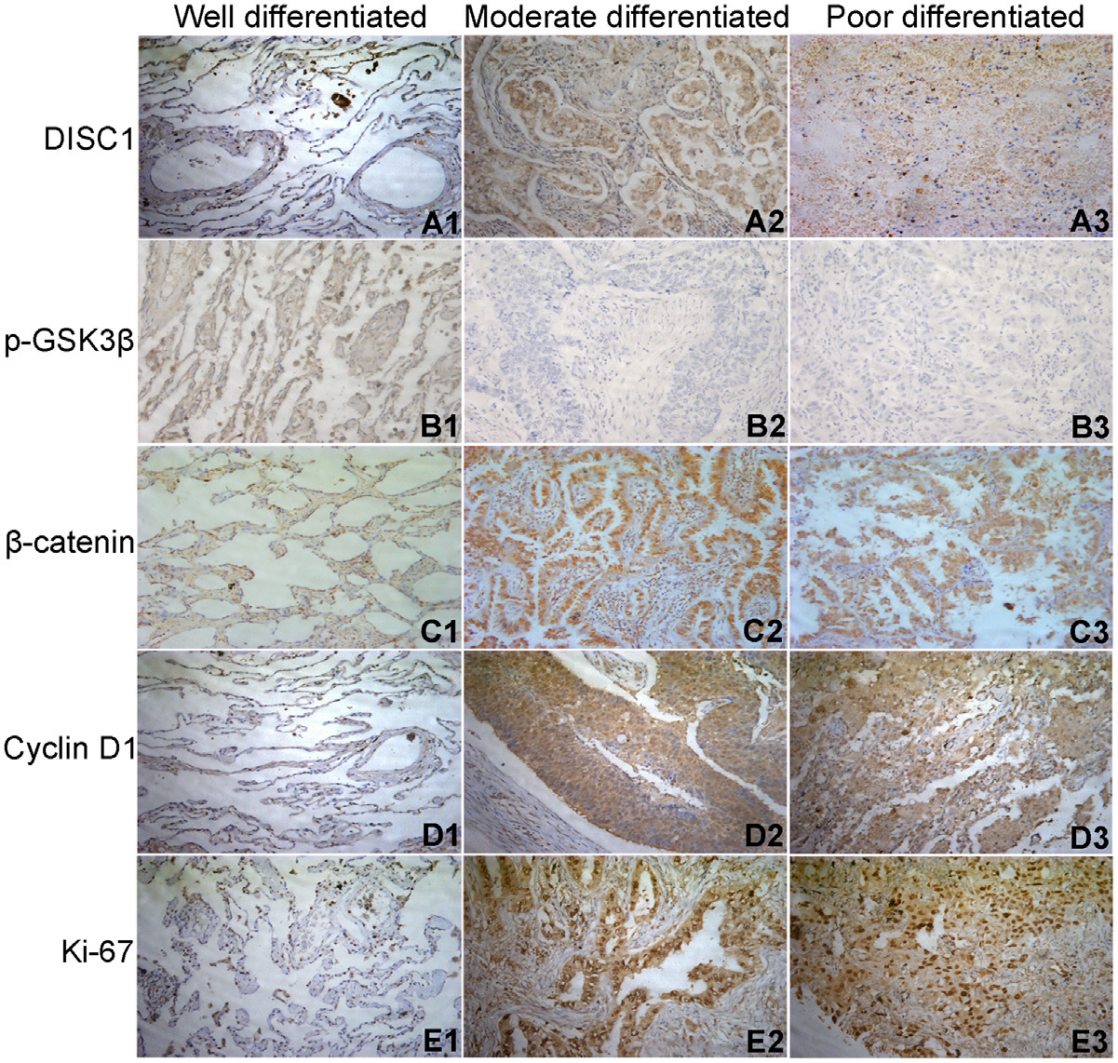
IHC staining of DISC1 **(A)**, p-GSK3β **(B)**, β-catenin **(C)**, Cyclin D1 **(D)**, and Ki-67 **(E)** in well- (1), moderately- (2), and poorly-differentiated (3) NSCLC tissues (200×).

DISC1 and the Wnt pathway molecules, p-GSK3β (Phosphorylation at Tyr216), β-catenin, Cyclin D1, and Ki-67, were measured in 140 NSCLC patient tissue samples via immunohistochemical (IHC) analysis. DISC1 and β-catenin were expressed in the cytoplasm and nucleus, p-GSK3β was prominently expressed in cytoplasm, and Ki-67 was expressed in the nucleus. DISC1, β-catenin, Cyclin D1, and Ki-67 levels increased from well- to poorly-differentiated NSCLC tissues (Figure 2), and increased with increasing tumor grade. DISC1 and p-GSK3β levels were negatively correlated, indicating that DISC1 might promote NSCLC development through negative regulation of GSK3β.

### Clinical relevance of DISC1 in NSCLC

**Figure 3:**
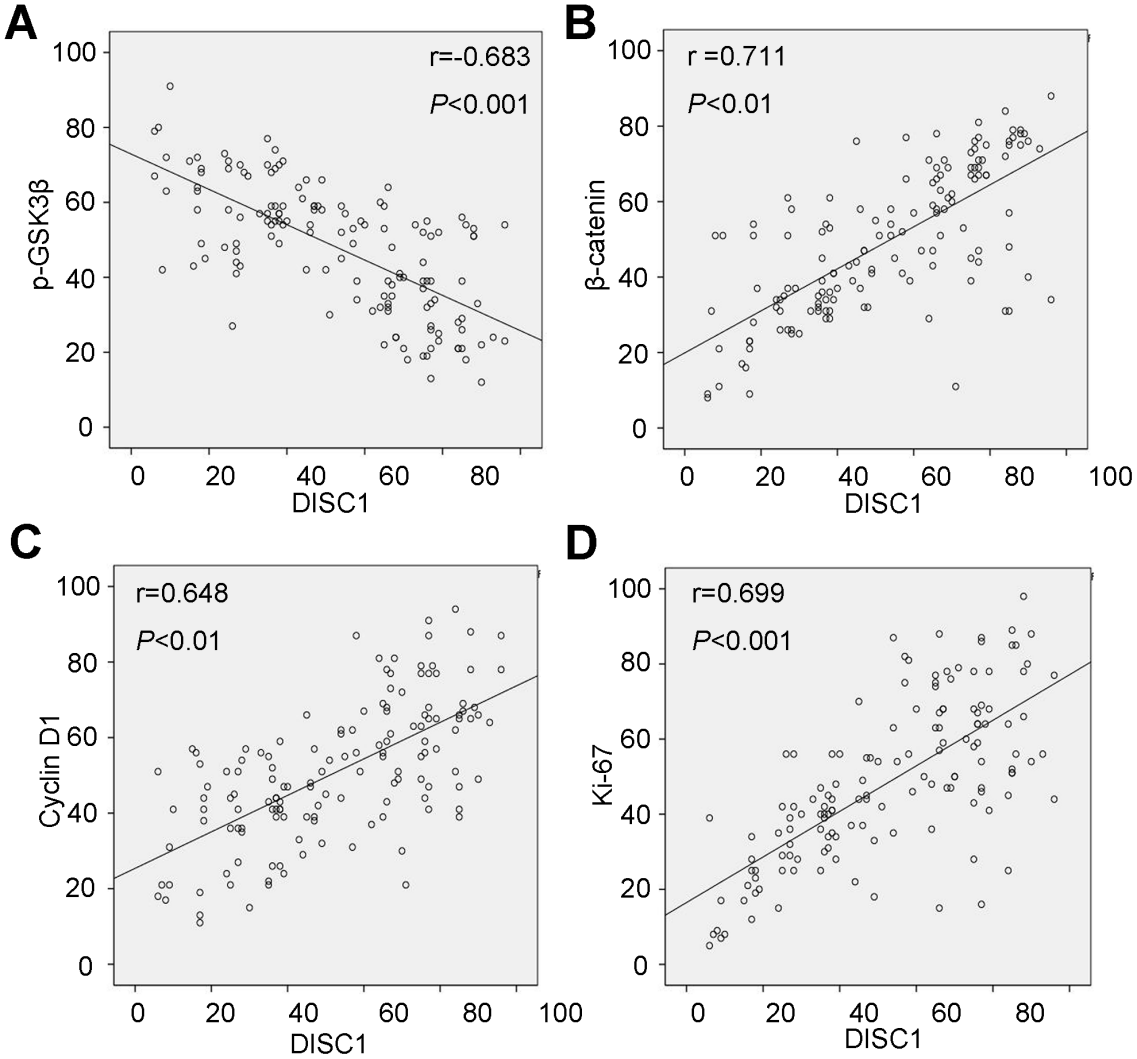
DISC1 expression correlated with that of p-GSK3β **(A)**, β-catenin **(B)**, Cyclin D1 **(C)**, and Ki-67 **(D)**.

Spearman’s rank correlation identified positive associations between DISC1 and β-catenin (r=0.711, *P*<0.01), Cyclin D1 (r=0.648, *P*<0.01), or Ki-67 (r=0.699, *P*<0.01) expression, and a negative correlation between DISC1 and p-GSK3β expression (r=-0.683, *P*<0.01) (Figure 3). DISC1 associations with clinicopathologic variables were evaluated by Pearson χ^2^ test. DISC1 level was considered high or low according to the cutoff value, for statistical analysis of DISC1 stain, 50% of malignant cells showing positive stain was used as a cutoff value to distinguish tumors with a low (<50%) or high (≥50%) level of expression [23]. High DISC1 expression was correlated with smoking status (*P*=0.049), tumor size (*P*=0.013), pathology grade (*P*<0.001), grade (*P*=0.020), lymph node metastasis (*P*=0.032), and Ki-67 (*P*<0.001), p-GSK3β (*P*<0.001)*, β-catenin* (*P*<0.001), *and Cyclin D1 expression* (*P*<0.001) (Table 1). There was no correlation with gender (*P*=0.800), age (*P*=0.375), clinical stage (*P*=0.123), or distant metastasis status (*P*=0.143).

**Table 1:** Associations between DISC1 expression and NSCLC patient clinicopathological parameters

Clinicopathological parameters	Total	DISC1		*P*-value
		Low (n=67)	High (n=73)	
Gender				
Male	112	53	59	0.800
Female	28	14	14	
Age				
<60	43	23	20	0.375
≥60	97	44	53	
Smoking status				
Yes	57	33	24	0.049*
No	83	34	49	
Tumor size (cm)				
≤3	60	36	24	0.013*
>3	80	31	49	
Clinical stage (TNM)				
T1+T2	125	57	68	0.123
T3+T4	15	10	5	
Pathology grade				
Poor	41	27	14	<0.001*
Mod	60	31	29	
Well	39	9	30	
Grade				
I+II	93	51	42	0.020*
III+IV	47	16	31	
Lymph node metastasis				
No	54	32	22	0.032*
Yes	86	35	51	
Distant metastasis status				
M0	105	54	51	0.143
M1	35	13	22	
p-GSK3β expression				
Low	66	13	53	<0.001*
High	74	54	20	
β-catenin expression				
Low	70	53	17	<0.001*
High	70	14	56	
Cyclin D1 expression				
Low	65	49	16	<0.001*
High	75	18	57	
Ki-67 expression				
Low	78	60	18	<0.001*
High	62	7	55	

*P<0.05 (Pearson χ^2^ test) was considered significant.

**Figure 4:**
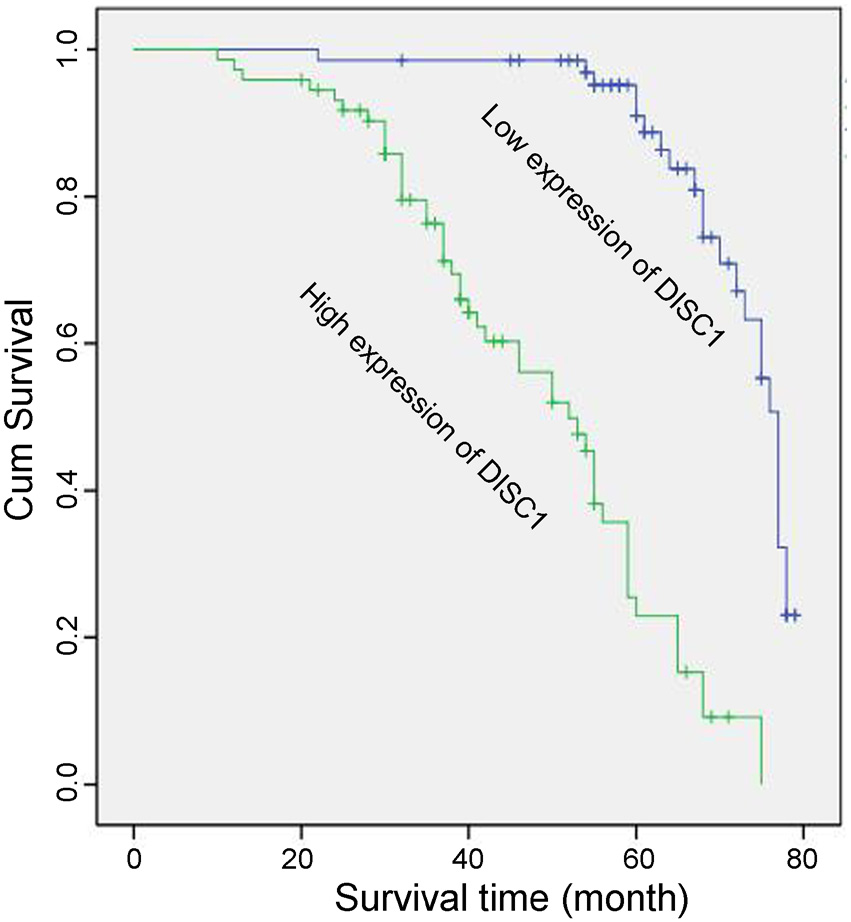
Kaplan-Meier survival analysis of DISC1 expression in NSCLC patients Cumulative overall NSCLC patient survival rate was associated with high (green line) or low (blue line) DISC1 expression.

Only 36% (26/73) of the 140 NSCLC patients analyzed survived from the high DISC1 expression group, while 66% (44/67) survived from the low expression group (Table 2), overall survival was calculated from the date of surgery to death or the date of last follow-up. DISC1 (*P*<0.01), tumor size (*P*=0.040), pathology grade (*P*<0.01) were correlated with patient survival status (Table 2). Cox’s proportional hazards model revealed that DISC1 (*P*<0.01) and Ki-67 (*P*=0.030) levels were independent prognostic factors for patient overall survival (Table 3). Kaplan-Meier analysis associated high DISC1 expression with poor patient survival (*P*<0.001; Figure 4).

**Table 2:** Univariate analysis of NSCLC patient clinicopathological parameters with respect to survival

Clinicopathological parameters	Total	Survival status		*P*-value
		Died	Alive	
Gender				
Male	112	55	57	0.673
Female	28	15	13	
Age				
<60	43	23	20	0.583
≥60	97	47	50	
Smoking status				
Yes	57	23	34	0.058
No	83	47	36	
Tumor size (cm)				
≤3	60	24	36	0.040*
>3	80	46	34	
Clinical stage (TNM)				
T1+T2	125	63	62	0.785
T3+T4	15	7	8	
Pathology grade				
Poor	41	12	29	<0.01*
Mod	60	27	33	
Well	39	31	8	
Grade				
I+II	93	42	51	0.107
III+IV	47	28	18	
Lymph node metastasis				
No	54	29	25	0.487
Yes	86	41	45	
Distant metastasis status				
M0	105	54	51	0.558
M1	35	16	19	
DISC1 expression				
Low	67	23	44	<0.01*
High	73	47	26	
p-GSK3β expression				
Low	66	35	31	0.498
High	74	35	39	
β-catenin expression				
Low	70	30	40	0.091
High	70	40	30	
Cyclin D1 expression				
Low	65	32	33	0.865
High	75	38	37	
Ki-67 expression				
Low	78	31	47	0.006*
High	62	38	23	

*P<0.05 (Pearson χ^2^ test) was considered significant.

**Table 3:** Cox regression analysis of potential prognostic factors with respect to NSCLC patient survival

	Hazard ratio	95% confidence interval	*P*-value
Tumor size (cm)	1.446	1.000-2.972	0.050
Pathology grade	1.169	0.991-1.978	0.057
DISC1 expression	2.498	1.140-5.798	<0.01*
Ki-67 expression	1.625	0.810-3.162	0.030*

*P<0.05 (Cox regression analysis) was considered significant.

### Effects of DISC1 on NSCLC cell cycle progression

**Figure 5:**
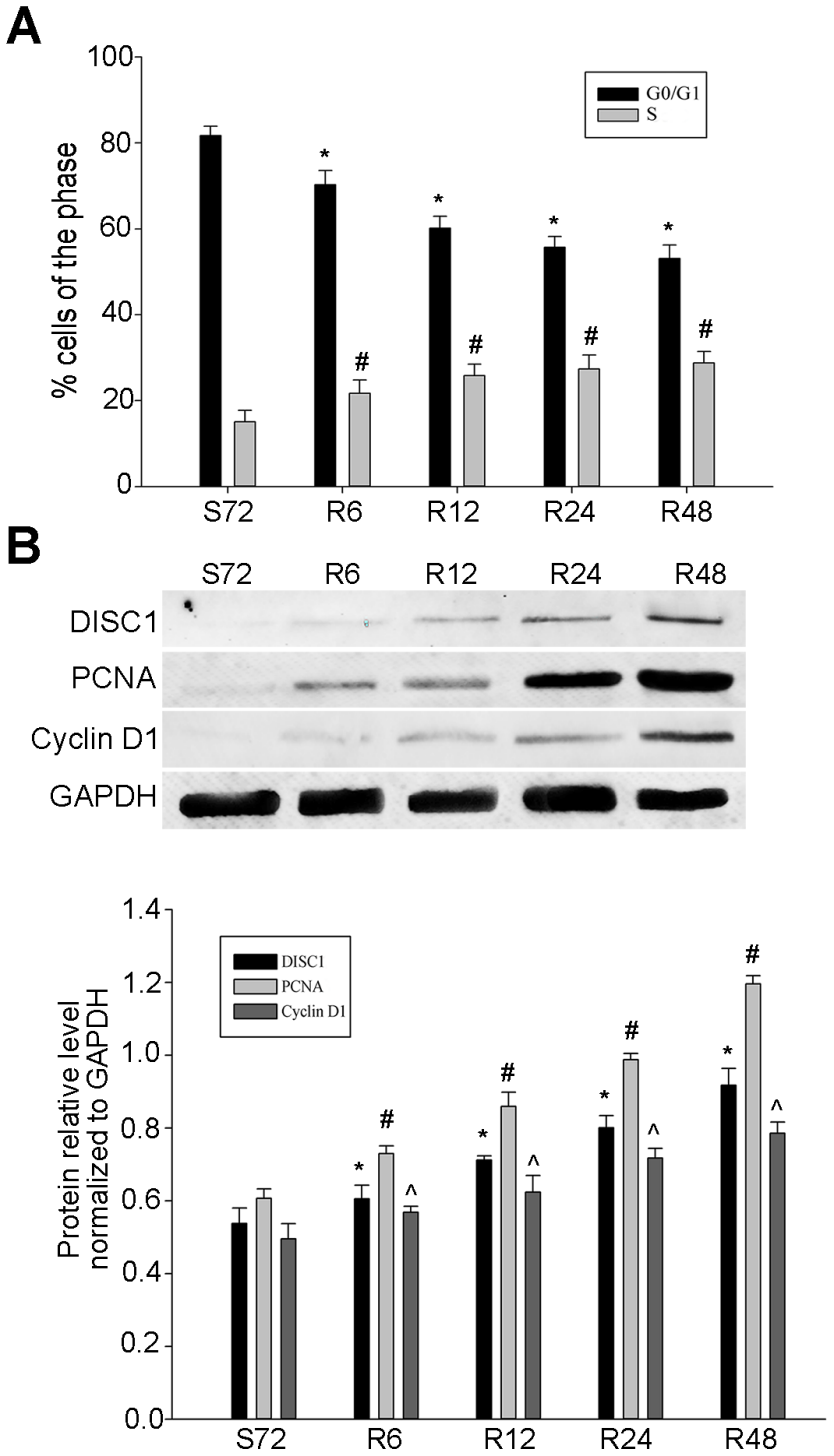
DISC1 promoted NSCLC cell proliferation A549 cell cycle progress was detected by flow cytometry **(A)** cells were synchronized at G1 phase after serum starvation for 72 h, and progressed into S phase with the addition of 10% FBS-supplemented medium for 6, 12, 24, or 48 h. PCNA and Cyclin D1 were detected by western blotting **(B)** PCNA and Cyclin D1 levels were analyzed (normalized to GAPDH). *, #, ^*P*<0.05, compared with cells (serum starved for 72 h). S: starvation; R: release.

DISC1 expression was correlated with that of the cell proliferation markers, PCNA and Ki-67, suggesting that DISC1 might regulate NSCLC cell cycle progression. Flow cytometry results showed that A549 cells, which exhibited the highest DISC1 levels of the three tested NSCLC cell lines, were blocked in G0/G1 phase after serum starvation for 72 h. When serum-free medium was replaced with 10% FBS-supplemented medium, cells were released from G1 phase and entered S phase (Figure 5A). The number of cells in G1 phase decreased from 91.50% to 52.46%, and those in S phase increased gradually from 5.14% to 29.78%. Western blotting analysis showed that DISC1, Cyclin D1, and PCNA levels increased after serum supplementation (Figure 5B).

### Effects of DISC1 silencing on NSCLC cell proliferation

**Figure 6:**
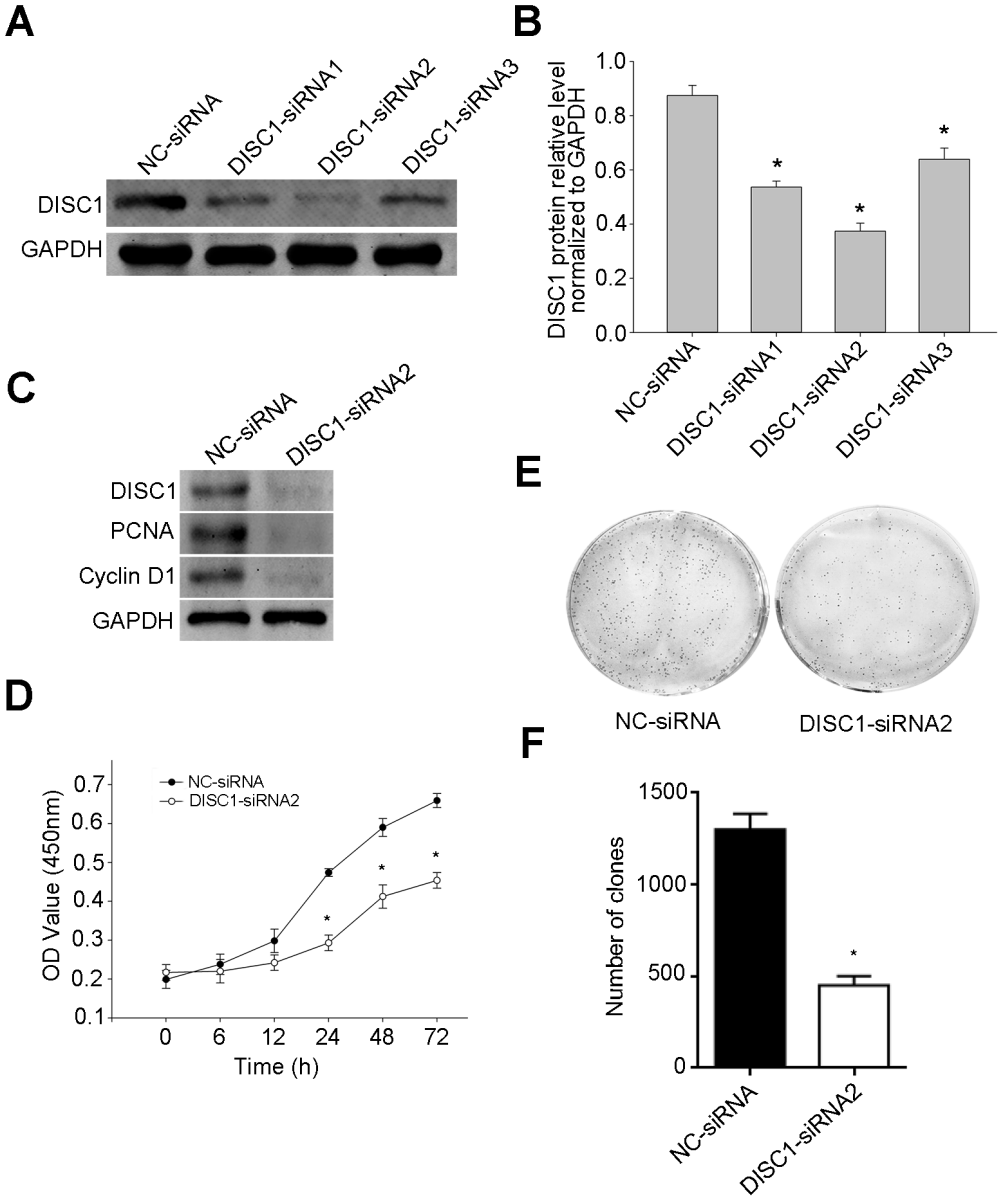
siRNA-mediated DISC1 silencing in NSCLC cells DISC1 was silenced using siRNAs (DISC1-siRNA1, DISC1-siRNA2 and DISC1-siRNA3) in A549 cells, as detected by western blotting **(A)** DISC1 knockdown by DISC1-siRNA1, DISC1-siRNA2, and DISC1-siRNA3 **(B)** PCNA and Cyclin D1 levels were detected by western blotting **(C)** DISC1-siRNA2-treated A549 cell proliferation as measured by CCK-8 assay **(D)** Colony formation by DISC1-siRNA2-treated A549 cells **(E)** The number of cell clones by DIC1-siRNA2-treated A549 cells **(F)** *P<0.05 compared NC-siRNA-treated cells.

DISC1-targeted siRNAs (DISC1-siRNA1, DISC1-siRNA2 and DISC1-siRNA3) and a negative control siRNA (NC-siRNA) were used to assess the impacts of DISC1 on cell proliferation. DISC1 levels decreased in DISC1-silenced A549 cells, as shown by western blotting (Figure 6A–6B). DISC1-siRNA2, which resulted in the greatest level of silencing, was employed in the following experiments. PCNA and Cyclin D1 levels decreased in cells treated with DISC1-siRNA2 (Figure 6C). DISC1 knockdown also reduced NSCLC cell growth rates as shown by CCK-8 assay (Figure 6D) and decreased cell colony formation (Figure 6E–6F).

### Correlation between DISC1 and p-GSK3β in NSCLC tissues

**Figure 7:**
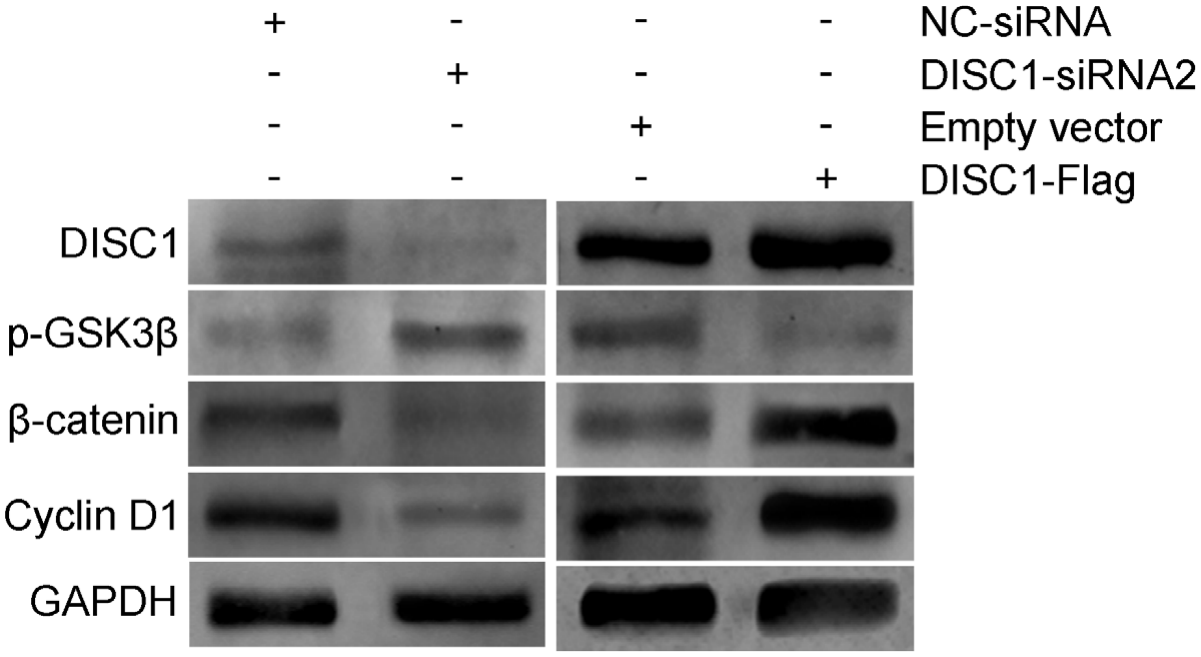
Effects of DISC1 expression on GSK3β/β-catenin signaling in NSCLC cells After DISC1 inhibition or upregulation in A549 cells using DISC1-siRNA2 or DISC1-Flag, respectively, DISC1, p-GSK3β, β-catenin, and cyclin D1 were detected via western blotting.

**Figure 8:**
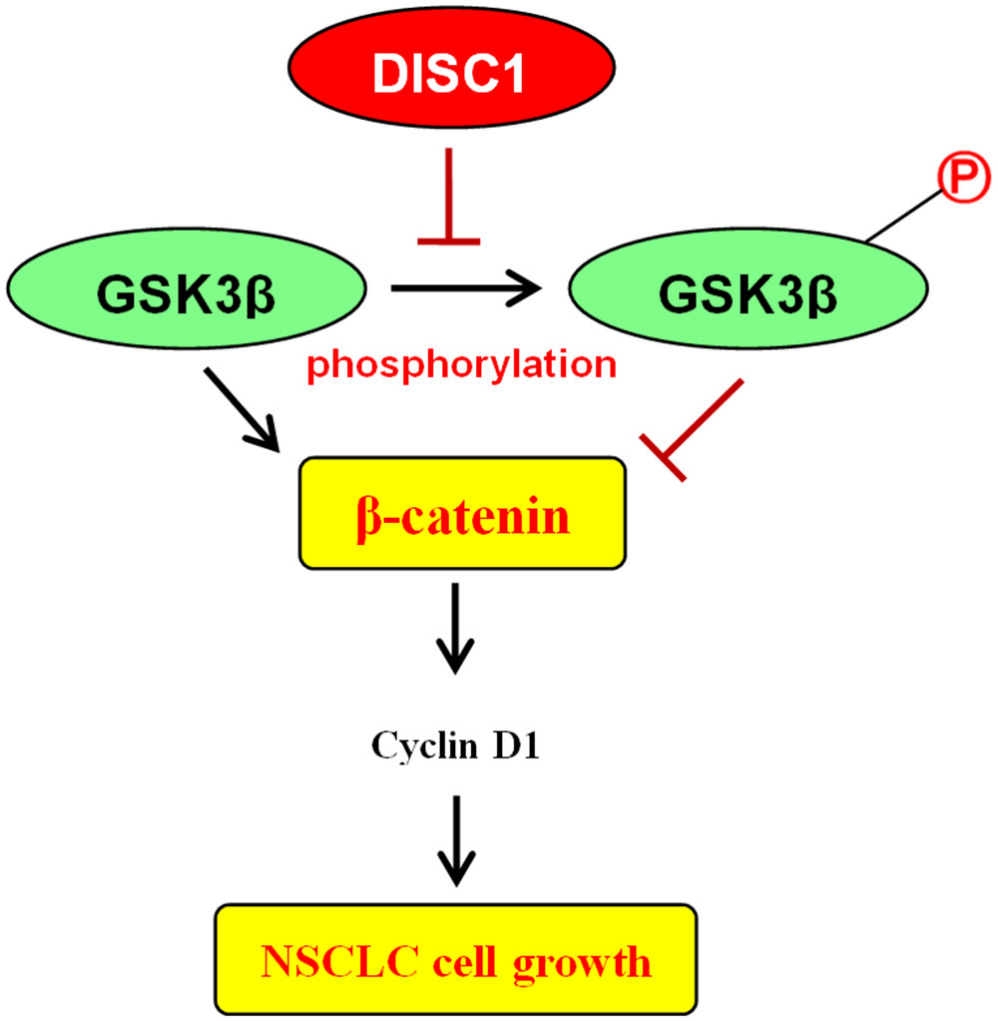
Hypothesized mechanism for DISC1 overexpression promotion of NSCLC cell proliferation

During brain development, DISC1 amino acids 195–238 regulate GSK3β/β-catenin/Wnt signaling by inhibiting GSK3β phosphorylation, which in turn reduces β-catenin phosphorylation and stabilizes β-catenin [14]. siRNA-mediated DISC1 silencing and a DISC1 over-expression vector (DISC1-Flag) were used to assess the effects of DISC1 on GSK3β in NSCLC. DISC1 knockdown increased p-GSK3β levels, and decreased β-catenin and Cyclin D1, while DISC1 upregulation produced the opposite results (Figure 7). Thus, we observed a negative correlation between DISC1 and p-GSK3β in NSCLC cell lines (Figure 8).

## DISCUSSION

Uncontrolled cell proliferation is a major factor in lung cancer development and progression [24]. A more complete understanding of proliferation mechanisms is necessary for the development of novel therapeutics that target uncontrolled cell growth. Our results showed that DISC1 overexpression in NSCLC tissues correlated with patient clinicopathologic factors and predicted poor prognosis, and high DISC1 expression promoted NSCLC cell proliferation. These results indicated that DISC1 might be a novel oncogene, and could promote NSCLC progression.

DISC1 was first associated with a variety of brain disorders, including schizophrenia, mood disorders, and autism [10–12]. DISC1 is an important neural progenitor cell proliferation regulator that positively modulates canonical Wnt signaling by inhibiting GSK3β catalytic activity, which activates β-catenin [14]. We found that DISC1 was overexpressed in NSCLC patient tissues, and hypothesized that it might promote NSCLC cell proliferation. IHC analyses demonstrated that DISC1 expression in NSCLC patient tissues correlated with that of p-GSK3β, β-catenin, Cyclin D1, and Ki-67, and was associated with clinicopathologic variables. Cox’s proportional hazards model indicated that DISC1 could be an independent prognostic factor for NSCLC patient survival, and Kaplan-Meier analysis showed that DISC1 overexpression predicted poor survival. Finally, a serum starvation and release assay indicated that DISC1 promoted A549 cell proliferation and might play a role in cell cycle progression.

Neurological studies reported that DISC1 directly interacts with GSK3β, and suggested that GSK3β promotes β-catenin phosphorylation and ubiquitylation-mediated β-catenin degradation in the proteasome [16–19]. However, DISC1 inhibits GSK3β activity by reducing β-catenin phosphorylation and stabilizing β-catenin during brain development, which in turn activates transcription of β-catenin target genes, including Cyclin D1 [20]. Whether or not DISC1 activates Wnt/β-catenin signaling through GSK3β inhibition in NSCLC remains unknown. Our results indicated that DISC1 might act as an oncogene in NSCLC development through Wnt/β-catenin signaling, and high DISC1 expression correlated with decreased GSK3β phosphorylation. Additionally, DISC1 knockdown increased p-GSK3β expression and decreased that of β-catenin and Cyclin D1, while DISC1 upregulation produced the opposite results. In summary, we identified a novel role for DISC1 as a potential NSCLC oncogene. Our study suggests that DISC1 might promote NSCLC cell proliferation through GSK3β/β-catenin signaling, and could be a novel anti-NSCLC therapeutical target.

## MATERIALS AND METHODS

### Patient tissue samples

Eight paired NSCLC and adjacent non-tumor tissues were collected immediately after surgical removal and stored at -80°C for western blot analysis. Additionally, 140 NSCLC specimens, along with patient demographic information and clinical data (Table 1), were obtained from the Department of Pathology, Affiliated Hospital of Nantong University between 2008 and 2013 for IHC analysis. Informed consent was obtained from all study participants. The Ethics Committee of the Affiliated Hospital of Nantong University provided permission to use tissue sections for research purposes.

### NSCLC cell lines and cultures

NSCLC cell lines (A549, H1299, and SPCA-1) were obtained from the Institute of Cell Biology, Chinese Academy of Sciences. Cells were cultured in RPMI 1640 medium (Gibco, USA) containing 10% fetal bovine serum (FBS) at 37°C with 5% CO_2_ [23].

### Western blotting and immunohistochemistry

Western blotting, and IHC analysis and evaluation were performed as described previously [23, 25–26]. Antibodies used were: anti-DISC1 (1:500 dilution; Santa Cruz Biotechnology, USA), anti-p-GSK3β (Tyr279/Tyr216) (1:500 dilution; Santa Cruz Biotechnology), anti-β-catenin (1:500 dilution; Santa Cruz Biotechnology), anti-Cyclin D1 (1:500 dilution; Santa Cruz Biotechnology), anti-PCNA (1:1000 dilution; Santa Cruz Biotechnology), and anti-GAPDH (1:3000 dilution; Sigma-Aldrich, USA).

### siRNAs, plasmid and transfection

siRNAs targeting DISC1 and negative control siRNA (NC-siRNA) were purchased from GenePharma Co., Ltd (China) (Table 4), and a DISC1 over-expression plasmid (DISC1-Flag) (GeneCopoeia, China) was also used. siRNAs and plasmid were transfected into NSCLC cell lines using Lipofectamine 2000 (Life Technologies, USA) according to the manufacturer’s instructions.

**Table 4:** DISC1-targeting siRNAs

siRNA name		Sequence (5'–3')
DISC1-siRNA1	Sense	GCUGAGACGUUACAACAAAdTdT
	Antisense	UUUGUUGUAACGUCUCAGCdTdT
DISC1-siRNA2	Sense	GCCAUAUCAGGAAACCAUUdTdT
	Antisense	AAUGGUUUCCUGAUAUGGCdTdT
DISC1-siRNA3	Sense	GGAAGCUUGUCGAUUGCUUdTdT
	Antisense	AAGCAAUCGACAAGCUUCCdTdT

### Cell cycle assay

A serum starvation and release process was used for cell cycle synchronization. Briefly, A549 cells were cultured in RPMI 1640 medium without FBS for 72 h, and were then replaced by 10% FBS-supplemented medium. Cells were then fixed in 70% ethanol for 1 h at 4°C and incubated with 1 mg/mL RNaseA for 30 min at 37°C. Cells were stained with propidium iodide (PI, 50 μg/mL) (Becton Dickinson, USA) in PBS and 0.5% Triton X-100, and analyzed using a BD FACScan flow cytometer (Becton Dickinson, San Jose, USA) and the CellQuest acquisition and analysis program.

### Cell proliferation assay

Cell counting kit-8 (CCK8) assay was performed to evaluate cell proliferation. A549 cells were seeded into 96-well cell culture cluster plates (Corning, USA) at 2×10^4^ cells/well, cultured overnight, and then transfected with DISC1-siRNA2 and negative control siRNA. CCK-8 reagents (Dojindo, Japan) were added to each well at different time points, and wells were incubated for an additional 2 h at 37°C in the dark. Absorbency was measured at 450 nm (reference 650 nm) with a microplate reader (Bio-Rad, USA). Experiments were repeated at least three times.

### Colony formation assay

Transfected A549 cells were seeded into 6-well cell culture cluster plates (Corning) at 200 cells/well, and incubated at 37°C with 5% CO_2_ for 10 d. Surviving colonies (>50 cells/colony) were stained with 0.5% crystal violet and counted.

### Statistical analysis

SPSS 19.0 software was used for statistical analysis. DISC1 expression and clinicopathological features were analyzed using the Chi-square (χ^2^) test. Kaplan-Meier curves were constructed and Log-rank test was performed to analyze survival data. Multivariate analysis was performed using Cox’s proportional hazards model. The risk ratio and its 95% confidence interval were recorded for each marker. *P*<0.05 was considered statistically significant. All values were expressed as means ± SD. Each experiment was repeated at least three times.
